# Factors associated with loneliness in Latin-American family care partners during the COVID-19 pandemic

**DOI:** 10.3389/fpsyt.2024.1286141

**Published:** 2024-11-12

**Authors:** Tomas Leon, Thamara Tapia-Munoz, Andrea Slachevsky, Bárbara Costa Beber, Fernando Aguzzoli, Carla Nubia, Mireya Vilar-Compte, Pablo Gaitan-Rossi, Loreto Olavarria, Loreto Castro, Alejandra Pinto, Tania Guajardo, R. Emilia Grycuk, Yaohua Chen, Iracema Leroi, Brian Lawlor, Claudia Duran-Aniotz, Roger O’ Sullivan, Claudia Miranda-Castillo

**Affiliations:** ^1^ Memory and Neuropsychiatric Centre (CMYN) Neurology Department, Hospital del Salvador and Faculty of Medicine, University of Chile, Santiago, Chile; ^2^ Global Brain Health Institute, Trinity College Dublin, Dublin, Ireland; ^3^ Millennium Institute for Care Research, Santiago, Chile; ^4^ Department of Behavioural Science and Health, University College London, London, United Kingdom; ^5^ Millennium Nucleus on Sociomedicine, Santiago, Chile; ^6^ Neuropsychology and Clinical Neuroscience Laboratory (LANNEC), Physiopathology Department - Institute of Biomedical Sciences (ICBM), Neuroscience and East Neuroscience Departments, Faculty of Medicine, University of Chile, Santiago, Chile; ^7^ Servicio de Neurología, Departamento de Medicina, Clínica Alemana Universidad del Desarrollo, Santiago, Chile; ^8^ Geroscience Center for Brain Health and Metabolism (GERO), Faculty of Medicine, University of Chile, Santiago, Chile; ^9^ Speech, Language and Hearing Sciences Department, Graduate Program in Rehabilitation Sciences, Federal University of Health Sciences of Porto Alegre, Porto Alegre, Brazil; ^10^ Universidad Iberoamericana, Mexico City, Mexico; ^11^ Department of Public Health, Montclair State University, Little Falls, NJ, United States; ^12^ Faculty of Medicine, department of psychology, Universidad Mayor, Santiago, Chile; ^13^ Department of Psychiatry, School of Medicine, Trinity College Dublin, Dublin, Ireland; ^14^ Geriatric Department, Université de Lille, Lille, France; ^15^ Latin American Brain Health Institute (BrainLat), Universidad Adolfo Ibáñez, Santiago, Chile; ^16^ Center for Social and Cognitive Neuroscience, Universidad Adolfo Ibáñez, Santiago, Chile; ^17^ Institute of Public Health in Ireland, Dublin, Ireland; ^18^ The Bamford Centre for Mental Health and Wellbeing, Ulster University, Coleraine, United Kingdom; ^19^ Faculty of Nursing, Universidad Andres Bello, Santiago, Chile; ^20^ Millennium Institute for Research in Depression and Personality, Santiago, Chile

**Keywords:** loneliness, family care partners, Latin America, COVID - 19, family caregivers

## Abstract

**Background:**

COVID-19-related restrictions led to an increase in overall loneliness and social isolation. Before the pandemic, care partners reported higher levels of loneliness and higher loneliness prevalence compared to non-care partners. Because of the spread and severity of the infections, and the access to support spread, we expect a different impact of the COVID-19 pandemic on LATAM care partners.

**Objectives:**

To describe the loneliness levels of LATAM caregivers and to identify socioeconomic and health factors associated.

**Design:**

An international online cross-sectional survey for care partners, embedded within the ‘Coping with Loneliness and Isolation during COVID-19’ (CLIC) Study conducted between June 2020– and November 2020.

**Setting:**

We analysed data from 246 family care partners living in Latin American countries (46% Mexico, 26% Chile,18% Brazil, and 10% from Argentina, Peru, Venezuela, Panama, Guatemala y Costa Rica).

**Measurements:**

We assessed loneliness using the 6-items of De Jong Gierveld loneliness Scale. We described the levels of overall, emotional, and social loneliness pre and during Covid, and reported the distribution of care partners who improved, worsened or maintained their levels of loneliness. Moreover, we used longitudinal multiple linear regression models with bootstraps errors of 1,000 iterations to identify factors associated with the levels of overall, emotional, and social loneliness during the pandemic.

**Results:**

Participants were mostly women, 50 years and older, in a partnership, highly educated and with finances meeting their needs, with good to excellent physical and mental health. Among the total of care partners, 55% perceived higher overall loneliness, 56% higher emotional loneliness, and 21% higher social loneliness during the pandemic in comparison with pre-COVID-19 levels. Perceived mental health was associated with the overall, emotional, and social loneliness.

**Conclusions:**

Regardless of their living and health situation, during the pandemic, loneliness increased in all groups of care partners. These should be taken in consideration when planning public health approaches for crises such as pandemics or other large-scale disruptive events.

## Introduction

Loneliness is often described as an unpleasant feeling that comes from the desire for a different social life or closer connections (1, 2). Weiss (3) identified two components of loneliness: emotional and social loneliness. Emotional loneliness is associated with the perceived absence or loss of an intimate connection, while social loneliness arises from a perceived lack of a social network (4–9). Research often focuses on overall loneliness (5, 10). Chronic loneliness, characterized by a high frequency and intensity, can have serious health consequences, including sleep disorders, depression, heart disease, dementia, and ultimately reduced life expectancy (10–12).

Loneliness is a steady feature with individual differences, which means, there are people more susceptible to feeling lonely during their lifespan. In the general population, people most at risk of loneliness are women, young adults or the oldest (80 + years), with a low level of education, low household income, living alone, experiencing pain, or living with a disease (10). However, specific individual or contextual factors can also trigger an increase in loneliness levels even for those with a low tendency for loneliness.

Globally, the COVID-19 (SARS-CoV-2) pandemic presented a significant public health challenge (13). Between 2020 and 2021, to curb the spread of COVID-19, many governments implemented various public health measures such as physical distancing, community lockdowns, closures of schools, workplaces, and public facilities, restrictions on large gatherings, and reduced use of public transport (14). Due to the long periods of confinement and physical restrictions, there was a growing concern about the health impact of social isolation and loneliness (15). At the start of the physical distancing measures, reports of loneliness in the general population significantly increased compared to pre-pandemic levels (16–19). For instance, the Coping with Loneliness, Isolation and COVID-19 (CLIC study) for the general population, from where the present study collected information, reported that among the 20,000 participants from over 100 countries, the prevalence of severe loneliness in adults 18 years old and older went from 6% before the pandemic to 21% during Covid-19 (15). Another study conducted in Japan reported that loneliness increased among younger and older adults, but the change was more intense and detrimental among younger adults (20). Finally, in the UK, four classes of loneliness trajectories were described, with levels of loneliness ranging from low to high at baseline. In the group with the highest levels, loneliness increased during the first year of the pandemic, while it decreased in the group with the lowest levels (16). Women, younger adults, with low income and economically inactive, or with mental health conditions were more likely to be in the highest-level loneliness group (16).

Before the pandemic, care partners reported higher levels of loneliness and lack of social support compared to non-care partners (21–24). However, findings have been inconsistent (23). During the pandemic, English-speaking caregivers experienced an increase in general, emotional and social loneliness and social isolation compared to pre-pandemic levels (25, 26). Moreover, most care partners of people with intellectual disability experienced the highest emotional and social loneliness during the pandemic (26). In the US, in a subsequential mixed method study, out of 82 care partners in Utah, 76.7% reported feelings of loneliness during the pandemic, and 21.9% mentioned they felt lonely every day. The care partners stated that their burden increased because they were their care recipient’s single social interaction and had to permanently provide support to their social needs (27).

The COVID-19 pandemic impacted Latin America (LATAM) especially hard, at one point, up to 25% of the global infections occurred in the region (28). During the first wave, eight of the ten countries with higher mortality were from LATAM (28). Because of their living conditions and the accessibility to services, it is expected that the impact on loneliness may differ from other regions (28, 29). Yet, data from LATAM, a region comprising nearly 9% of the world’s population, have not been reported.

There are several knowledge gaps regarding the impact of the pandemic on loneliness in LATAM care partners. In consequence, the aims of this study were 1) to describe the mean level of overall, emotional and social loneliness among LATAM care partners during the COVID-19 pandemic, and 2) to identify sociodemographic risk factors for loneliness mean levels during the COVID-19 pandemic.

## Methods

### Study design

This was a cross-sectional study embedded within the ‘Coping with Loneliness, Isolation and COVID-19’ (CLIC) project, an international online self-administered survey, conducted between June 2nd, 2020– and November 16th, 2020. CLIC aimed to analyse the changes in loneliness and social isolation during the COVID-19 pandemic among adults 18 years globally. The survey was developed through consensus by the International Loneliness and Isolation Research Network (I-LINK), and it is described elsewhere (15). The CLIC study was approved by the Ethics Committee of Ulster University (RG3) on 15th May 2020. Additional ethical approval was obtained in each country when the local regulation required it. All participants gave informed consent.

The survey’s recruitment was coordinated by a nominated investigator for each participating country, who accessed potential participants through the email lists and websites of public or voluntary sector organizations supporting family or informal care partners of people with brain health conditions and snowballed the survey link through social media networks such as Facebook and Twitter. To maximize uptake, each investigator distributed the survey at least twice during the data collection period, with a minimum of four weeks between distributions. Participants were internet users, and aged eighteen and older. Given that the participants were volunteers recruited online, we acknowledge a representation issue in this sample. At the same time, very few studies have reported the loneliness levels among care partners living at countries in LATAM.

### Study participants

Within the CLIC survey, the CLIC-Global Care Partners Sub-Study included 5,236 participants who self-classified as informal care partners (also called family caregivers meaning those who give care to family or friends usually without payment) of people with enduring physical (n=3,234) or brain health-related conditions (n=2,379 dementia; n=855 mental ill-health). Most of the care partner respondents were from USA and Canada (45%) and Europe (33%).

**Figure 1:**
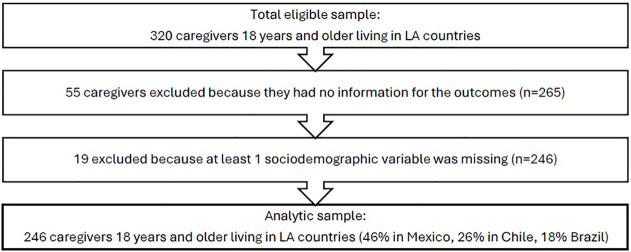
Study flowchart.

Participants who self-identified as care partners residing in LATAM (n=320; 7% out of the total care partners sample) were considered for the current study. Of them, 55 (17% of the initial sample) were excluded because they had missed three or more items about social and emotional loneliness. Additionally, 24 participants (7.5%) were excluded because of missing data in the covariates. Out of the final sample of 246 caregivers, 68 (27.64%) cared for PLWD and 175 (71.14%) cared for people with other enduring conditions, 3 care partners (1.22%) did not disclosure the condition of the care recipient but were included in the analysis because of the availability of sociodemographic and loneliness information. In addition, 46% were care partners from Mexico, 26% from Chile and 18% from Brazil (see **Figure 1**). See the complete list of countries in the **Supplementary Materials Table S1**.

### Study variables

The online survey encompassed 129 questions, including sociodemographic factors, measures of care partners’ burden, loneliness, social isolation, and general health, relating to their status pre and during the COVID-19 pandemic. Information from pre-COVID-19 used as baseline was retrospectively collected by asking the participants to remember the status of their physical and mental health, loneliness, and social isolation.

### Measurements

#### *Outcomes:* overall, emotional, and social loneliness

The questionnaire included R-UCLA loneliness items and the De Jong Gierveld Scale. Because National Surveys of Ageing measure loneliness using items from the UCLA Loneliness Scale, we selected it to report overall loneliness to allow comparability. Emotional and social loneliness were measured using the De Jong Gierveld Loneliness Scale. Loneliness was measured twice in the questionnaire. At the beginning, participants answered about their pre-COVID-19 loneliness, and at the end, they answered about their current levels of loneliness (during COVID-19). We reported both measurements but only modelled during COVID-19 loneliness due to the risk of recall-biased results in the pre-COVID-19 measurement.

#### Overall loneliness

Loneliness was assessed using the revised 3-item UCLA loneliness scale (30). The three items ask how often people feel “left out”, “lack of companionship” and “isolated”. The response scale was from hardly ever (1), some of the time (2), and often (3), providing a total score between 3 and 9 with higher scores denoting a higher level of loneliness.

#### Emotional and social loneliness

Emotional and social loneliness were measured using the 6-items version of the De Jong Gierveld Scale (5). The three items that measured emotional loneliness were ‘emptiness’, ‘miss people around’, and ‘rejected’, while the three items that measured social loneliness were ‘plenty I can rely on’, ‘many people I trust’, and ‘enough people I feel close to’. The total scores for both subscales ranged from 0 to 3, where 0 represents the least lonely and 3 represents the loneliest participants.

### Independent variables

#### Sociodemographic and health characteristics

Based on the original CLIC study (15) and the factors previously described in association with loneliness (10, 31) we selected potential risk and protective factors for the study. We used self-classified binary gender (men or women). A small number of participants chose the “other” or “prefer not to say” options, so they were recoded as missing because of power analysis considerations. Age was measured in 13 groups in the questionnaire (18-24, 25-29, 30-34, 35-39, 40-44, 45-49, 50-54, 55-59, 60-64, 65-69, 70-74, 75-79, 80-99). However, considering the sample size of our study and distribution, we used age in five groups (a) 18-34, b) 35-44, c) 45-54, d) 55-69, e) 70 and over). Marital status referring to people legally married or in relationships was categorised in two groups 1 “married/with partner/cohabiting” and 0 “never/not currently”. Education was measured using four categories “elementary” “diploma” “degree” and “postgraduate” studies. Considering the number of observations within all the categories (7% elementary, 5% diploma, 31% degree, and 56% postgraduate), we recoded the variable into two groups “postgraduate studies” and “less than postgraduate studies”. Participants were also asked about their living situations. We created the variable living alone where 1 refers to people living alone and 0 people living with others (partners, family, or friends). Among the 320 LATAM care partners included in the initial analytic sample, only 6 of them self-classified as living in rural areas. Because of the lack of observations, the variable was not included in the multiple regression analysis.

#### Classification of family care partners

Participants selected the diagnosis of their care recipients from four categories: care partners of people living with dementia (PLWD), mental health problems, intellectual disabilities, chronic physical disabilities, other disabilities, or dual disability issues. Because of the sample and the mental health issues largely described for caregivers of PLWD, the variable was also recoded into two groups, care partners of PLWD and non-dementia care partners. Additionally, we used the type of kin relationship within the care dyads classified into three groups: spouses (1), family members (2), and non-relatives (3).

#### Social isolation

Social isolation changes were measured by modifying the response scale of the six-item Lubben Social Network Scale (LSNS-6) (15). The LSNS-6 includes three items about family contacts and three items about contact with friends. To assess the changes from pre to during the pandemic, the participants answered if isolation was “about the same” (0), “more than usual” (+1), or “less than usual” (−1). We created the categorical variable “worse social isolation” where 1) was “worse social isolation” encompassing those who answered, “more than usual” and 0) “as usual or better social isolation” including the participants who used the options “less than usual” or “about the same”.

#### Perceived physical and mental health

The participants self-rated their physical and mental health as poor to excellent. We recodified their answers as binary dummy variables. Responses of “excellent”, “very good” or “good” were categorized as 1 “Good Ph/M health” and fair or poor as 0 “Poor to fair Ph/M health”.

#### Care partners’ burden

Burden was measured using the question “*How often do you feel burdened by the caring role*?”. The participants answered in a scale of five categories: 1) “never”, 2) “rarely”, 3) “sometimes”, 4) “quite frequently”, and 5) “nearly always”. Considering the number of observation available, we added burden as an ordinal variable into the models, where closer the scores to five, higher level of burden.

### Statistical analysis

#### Descriptive analysis

We described the sociodemographic and health characteristics by gender using Chi2 and Kruskal Wallis analysis. Moreover, we described the levels of overall, emotional, and social loneliness, the number of care partners with the same level of loneliness before and during the pandemic, and the percentage of them who increased or decreased their levels.

#### Association analysis

First, we tested different exposures and covariates using single regression models (see **Supplementary Tables S2**-**S4** in the **Supplementary Material**). The covariates “gender”, “age” and “social isolation” of the care partners were included regardless of the significance level in the bivariate analysis, while for the rest, only those with a p-value<0.05 at least in the follow-up were included in the final model.

We used the mean level of overall, emotional, and social loneliness during COVID-19 and performed a multiple linear regression using the STATA command “reg”. Given that loneliness scales (overall emotional, and social loneliness) produced a non-normally distributed discrete variable (see **Supplementary Figure S1** in the **Supplementary Materials**), we used bootstrap errors with 1000 iterations to estimate more precise confidence intervals. We chose the final models by balancing the goodness of fit (AIC) and the adjusted total percentage of variance explained (R2). The variables type of care partners (two or six groups), kin relationships, alcohol consumption, smoking, sleeping hours, anyone who died during Covid, Covid-related deaths, hospitalisation due to Covid, and physical perceived health were only associated in the bivariate analysis; therefore, not included in the models.

We determined factors associated with the caregiver’s overall loneliness levels during the pandemic (Equation 1), the caregiver’s emotional loneliness during the pandemic (Equation 2), and the caregiver’s social loneliness during the pandemic (Equation 3). We used conventional levels to consider a statistically significant association with a p-value lower than 0.05 and confidence intervals without the null value.


(1) $$\begin{array}{l} Y_{1}=\beta_{0}+\beta_{1}*\%>Age\;{\left(\%\right)}_{i}+\beta_{2}*\%Women_{i}+\beta_{3}*\%people\;with\;postgraduate\;education_{i}+\beta_{5}*Burden_{i}+\beta_{5}*perceived\;mental\;health_{i}+\beta_{6}*\Delta social\;isolation_{i}+\mu_{c} \end{array}$$

(2) $$Y_{I}=\beta_{0}+\;\beta_{1}*Age\;{\left(\%\right)}_{i}+\;\beta_{2}*\%\;Women_{i}\;+\beta_{3}*\%\;fairly\;well\;finances_{i}+\;\beta_{4}*\%\;poorly\;finances_{i}+\beta_{5}*\Delta \;mental\;health_{i}+\;\beta_{6}*\Delta \;social\;isolation_{i}+\;\mu_{c}$$

(3) $$\begin{array}{l} Y_{1}=\beta_{0}+\beta_{1}*Age\;{\left(\%\right)}_{i}+\beta_{2}*\%Women_{i}+\beta_{3}*\%people\;with\;postgraduate\;education_{i}+\beta_{4}*\\ Burden_{i}+\beta_{5}*\%good/excelente\;physical\;health_{i}+\beta_{6}*\%good/excelente\;mental\;health_{i}+\beta_{7}*\\ \Delta social\;isolation_{i}+\mu_{c} \end{array}$$

Y refers to the overall loneliness, emotional or social loneliness, respectively; and “i” refers to variables measured at the individual level. Cross-sectional linear regression models were used. Δ stands for variation between the period pre-COVID and during COVID.

## Results

### Care partners’ sociodemographic and health characteristics

Latin American family care partners were mainly women (79%), highly educated (44% had a degree and 56% had a postgraduate degree) and with finances that met their needs very to fairly well (88%), caring for a relative other than their spouse (87%) with disability due to dementia (22.22%), a physical illness (27.16%) or other conditions (23.87%). Eight percent of the care partners reported their care recipients having two different diagnosis. Men participants age was between 18 and 74 years, while women were up to 99 years old (see **Supplementary Materials**, **Supplementary Figure S2**). The age groups were recodified using five groups (the majority of the participants were fifty years and older (55%)) (**Table 1**).

**Table 1:** Socioeconomic characteristics of the participants.

	Men (21.14%)		Women (78.86%)		Total		p-value
	n	%	n	%	n	%	
Age							
18-34	12	23.08	30	15.46	42	17.07	0.280
35-44	5	9.62	36	18.56	41	16.67	
45-54	12	23.08	57	29.38	69	28.05	
55-69	21	40.38	61	31.44	82	33.33	
70+	2	3.85	10	5.15	12	4.88	
Marital status							
Never/not currently	22	42.31	82	42.27	104	42.28	0.996
Married/current partner/cohabiting	30	57.69	112	57.73	142	57.72	
Educational level							
Postgraduate	24	46.15	113	58.25	137	55.69	0.119
Other educational level	28	53.85	81	41.75	109	44.31	
Finances meet needs							
Very well	14	26.92	68	35.05	82	33.33	0.532
Fairly well	26	50	84	43.3	110	44.72	
Poorly	12	23.08	42	21.65	54	21.95	
Location							
City	44	86.27	181	93.3	225	91.84	0.090
Town	5	9.8	12	6.19	17	6.94	
Rural	2	3.92	1	0.52	3	1.22	
Kin relationship							
Spouse	3	5.77	23	11.86	26	10.57	0.384
Family relative	48	92.31	165	85.05	212	86.59	
Non-relative	1	1.92	6	3.09	7	2.85	
Diagnose of the care recipient							
Dementia	11	21.15	43	22.51	54	22.22	0.510
Physical issues	16	76.92	50	26.18	66	27.16	
Mental Health issues	5	9.62	29	15.18	34	13.99	
Intellectual issues	5	9.62	6	3.14	11	4.53	
Others	11	21.15	47	24.61	58	23.87	
Dual	4	7.69	16	8.38	20	8.23	

p-values came from linear regression models and Chi2 tests.

Most of the participants had an excellent to good physical and mental health and reported similar or better social isolation during the COVID-19 pandemic than before the pandemic. Men and women differed in their alcohol and smoking consumption, as well as the changes in their social isolation and the burden due to caring role (**Table 2**).

**Table 2:** Health characteristics of the participants.

	Men (21.14%)		Women (78.86%)		Total		
	n	%	n	%	n	%	
Someone died during the pandemic							
No	34	65.38	131	67.53	165	69.07	0.770
Yes	18	34.62	63	32.47	81	32.93	
Alcohol consumption							
Less than before	12	23.08	30	15.46	42	17.07	0.022
More than before	2	3.85	19	9.79	21	8.54	
About the same	20	38.46	44	22.68	64	26.02	
Don’t partake	18	34.62	101	52.06	119	48.37	
Smoking consumption							
Less than before	2	3.85	3	1.55	5	2.04	0.001
More than before	2	3.85	10	5.18	12	4.90	
About the same	9	17.31	4	2.07	13	5.31	
Don’t partake	39	75	176	91.19	215	87.76	
Sleeping hours							
Less than before	6	11.54	10	5.15	16	6.50	0.168
More than before	21	40.38	98	50.52	119	48.37	
About the same	25	48.08	86	44.33	111	45.12	
Perceived physical health							
Excellent/very good/good	45	86.54	155	79.9	200	81.30	0.275
Fair/poor	7	13.46	39	20.1	46	18.70	
Perceived mental health							
Excellent/very good/good	45	86.54	165	85.05	210	85.37	0.788
Fair/poor	7	13.46	29	14.95	36	14.63	
Δ Social isolation							
Better or equal	45	86.54	142	73.2	187	76.02	0.045
Worse	7	13.46	52	26.8	59	23.98	
Burden by caring role							
Never/rarely/sometimes	42	80.77	118	60.82	160	65.04	0.007
Frequently/Always	10	19.23	76	39.18	86	34.96	

p-values came from linear regression models and Chi2 tests.

### Levels of overall, emotional, and social loneliness and their self-perceived changes

**Table 3** depicts the levels of total, emotional, and social loneliness pre and during the COVID-19 pandemic and the distribution of the changes between measurements. During the pandemic, there was an increase in the loneliness mean levels of overall, emotional, and social loneliness. Accordantly, 55% of the care partners reported higher levels of overall loneliness during the pandemic while 56% of them reported higher levels of emotional loneliness. Surprisingly, 70% of the care partners reported the same level of social loneliness before and during the pandemic, while 21% perceived they had a higher level of social loneliness. There were no differences between care partners of PLWD and other health conditions. Based on Pearson correlations, pre and during COVID overall, and emotional loneliness had a moderate association (r=0.37, r=0.28, respectively), while the pre and during social loneliness had a high association (r=0.60).

**Table 3:** levels of overall, emotional, and social loneliness and their changes during the pandemic.

	Before the pandemic	During the pandemic
Overall loneliness (mean(sd))	4.06 (1.33)***	5.16 (1.84)***
Emotional loneliness (mean(sd))	0.84 (0.99)***	1.66 (0.98)***
Social loneliness (mean(sd))	1.93 (1.32)***	2.20 (1.23)***
Δ Overall loneliness	Freq.	%
The same	73	29.67
Higher loneliness	135	54.88
Lower loneliness	38	15.45
Δ Emotional loneliness		
The same	84	34.15
Higher loneliness	137	55.69
Lower loneliness	25	10.16
Δ Social loneliness		
The same	173	70.33
Higher loneliness	52	21.14
Lower loneliness	21	8.54

Δ stands for changes between measurements. The same is the % of people that perceived the same mean levels of loneliness. Higher loneliness is the % of people who perceived an increment in their mean levels during the pandemic. Lower loneliness is the % of people who perceived a decrease in their mean levels during the pandemic. ***p<0.001.

### Factors associated with total, emotional, and social loneliness among Latin American family care partners during the COVID-19 pandemic

**Table 4** displays the results of the multiple linear regression models for the mean level of overall, emotional, and social loneliness during the pandemic.

**Table 4:** multiple linear regression models for overall, emotional, and social loneliness (n=246).

	Overall loneliness			Emotional loneliness			Social loneliness		
	ß coef.	95% CI		ß coef.	95% CI		ß coef.	95% CI	
35-44	-0.112	-0.863	0.639	-0.422*	-0.839	-0.005	-0.054	-0.517	0.410
45-54	0.126	-0.542	0.793	-0.113	-0.469	0.242	-0.405	-0.864	0.053
55-69	0.268	-0.345	0.882	-0.014	-0.343	0.315	-0.300	-0.720	0.119
70 and over	-0.009	-1.303	1.285	-0.339	-1.028	0.350	-0161	-0.886	0.565
Gender	0.285	-0.257	0.827	0.239	-0.057	0.536	0.174	-0.208	0.556
Educational level	0.429	-0.044	0.903				0.490**	0.182	0.799
Burden	0.919***	0.451	1.388				0.205	-0.108	0.518
Finances met needs									
Fairly well	–			0.172	-0.091	0.435	–		
Poorly	–			0.428*	0.097	0.759	–		
Physical Health				–			-0.115	-0.491	0.261
Mental health	0.719*	0.045	1.394	0.454**	0.145	0.764	0.283	-0.055	0.620
Δ social isolation	0.850***	0.348	1.351	0.515***	0.267	0.764	0.257	-0.044	0.612
Constant	4.024***	3.348	4.700	1.231***	0.831	1.632	1.919***	1.441	2.398
Model specifications									
R2	0.171			0.157			0.089		
Adjusted R2	0.139			0.125			0.051		
AIC	968.131			665.095			798.702		
Chi2 (p-value)	53.04 (<0.001)			54.33 (<0.001)			30.32 (<0.001)		

*<0.05 **<0.01 ***<0.001. Ref category for age “18 to 34”. Ref category for agender “Men”. Ref category for educational level “Less than postgraduate education”. Ref category for mental health “Good/excellent”. Ref category for burden “never to sometimes”. Ref category for finances “Very well”. Ref category for worse social isolation “less or equal social isolation”. Models were conducting using bootstrap errors. 95% CI first column depicts min value; second column depicts max values.

The final model for overall loneliness during the pandemic considered age, gender, educational level, burden, perceived mental health, and the changes in social isolation. The factors accounted for 14% of the adjusted variance. Worse social isolation during the pandemic (β coef. = 0.850; 95% CI: 0.348, 1.351), frequent feelings of burden because of care duties (β coef. = 0.919; 95% CI: 0.451, 1.388), and poor mental health (β coef. = 0.719; 95% CI: 0.045, 1.394) were associated with higher overall loneliness during the pandemic. Marital Status and Kin relationship were also associated with loneliness in the bivariate analysis but excluded from the multilevel models due to their low contribution to the Goodness of fit. Visual analysis of the residuals showed an adequate goodness of fit for the model (see **Supplementary Materials**, **Supplementary Figure S3**).

The final model for emotional loneliness encompassed age, gender, whether finances met needs, perceived mental health, and the change in social isolation. It explained 13% of the emotional loneliness variance. According to conventional levels, finances poorly meeting participant’s needs was associated with higher levels of emotional loneliness (β coef. = 0.428; 95% CI: 0.097, 0.759). Similarly, family care partners who perceived a poor mental health (β coef. = 0.454; 95 CI: 0.145, 0.764) and had worse social isolation during the pandemic reported higher emotional loneliness (β coef. = 0.515; 95 CI: 0.267, 0.764). Finally, care partners who were between 35 and 44 years old had lower levels of emotional loneliness compared to those 18-34 (β coef. =-0.422; 95 CI: -0.839, -0.005).

Interestingly, age, gender, educational level, burden, perceived physical and mental health and the changes in social isolation accounted for only 5% of the total variance of social isolation during the pandemic. Participants with less than postgraduate education (β coef. = 0.490; 95 CI: 0.182, 0.799) had higher levels of social loneliness than those with postgraduate education.

## Discussion

To our knowledge, this is the first study exploring the levels of overall, emotional, and social loneliness during the COVID-19 pandemic among care partners of people with chronic conditions living in LATAM. Care partners in this study were primarily women 50 years and older, in a partnership, highly educated and with finances meeting their needs. They were caring for a non-spouse family member with a physical, mental, or cognitive disability other than dementia.

Our study found that care partners experienced higher levels of overall, emotional, and social loneliness during the COVID-19 pandemic. We call upon interpreting the pre-COVID loneliness levels with caution due to potential recall bias. Nevertheless, our results confirm what was observed in the general sample of the CLIC Global Care Partners Study (25, 26) and other studies conducted among the general population (25, 32)—showing higher levels of loneliness during the pandemic and no differences by region. Given that loneliness is considered a risk factor for depression (33, 34), these findings should raise concern about the mental health of care partners even during the post-social restrictive measures’ time. The mental health consequences might remain, especially if countries face a humanitarian crisis after the COVID-19 emergency (35).

Very few studies have described the levels of loneliness among family care partners of PLWD (21, 36), and other family care partners (37). Although in general, care partners of people living with dementia (PLWD) have shown worse mental health, including loneliness and burden, than care partners of people with other chronic conditions (38), in our study, the overall, emotional, and social loneliness did not differ between care partner of PLWD and other conditions. The recently published report of the CLIC Global Care Partners Study (n=3,930), where care partners of PLWD were one of the groups compared, found that the most affected groups of care partners were those taking care of relatives with intellectual disability and dual conditions (26). LATAM countries have been improving their health and social care systems to provide universal care and support people living in socially deprived conditions, However, it varies by country. Some LATAM countries are low-income, with a low public expenditure, and an important health gap, including a lack of hospital beds, and specialists (14). Additionally, as in the rest of the world, public and private systems were highly impacted by the pandemic, affecting usual care. All these factors might have decreased the level of support that people living with any long-term physical, cognitive, or mental health conditions received, indirectly affecting care partners’ mental health regardless of the care recipient’s diagnosis or condition (35).

Perceived mental health was associated with overall, and emotional loneliness. The protective role of a positive perception of mental health has been described for loneliness and other health outcomes (11, 39, 40). Moreover, even though, we did not report depressive symptoms or other mental health conditions, our results might reflect the previously described link between stress, anxiety, depression, and loneliness (31, 33, 41, 42).

The experience of burden due to care tasks was associated with overall loneliness. Previously, a study in Singapore reported that care partners moderately connected but lonely reported higher levels of burden (43). Noteworthy, the analysis of the English-speaker CLIC participants showed an increased burden among participants with severe emotional loneliness.

Educational level was the only factor associated with social loneliness regardless of age, gender, self-reported physical and mental health, and the changes in social isolation. Even though our sample was conformed mostly of highly educated people, those with a postgraduate degree had a significantly lower level of overall loneliness. Education level has been linked to overall, emotional and social loneliness (24, 36, 44); and it has been used before as a proxy for socioeconomic status. High socioeconomic status can be associated with loneliness because it provides positive living conditions, including the possibility for social connections and leisure activities (45, 46). In our study, we also used an additional measure of economic status, asking how well care partners’ finances met their needs. The finances were only statistically significantly associated with emotional loneliness and were not included in the overall and social loneliness models. We hypothesize that people with a postgraduate degree had more skills to cope with the economic and other consequences of the pandemic, being able to switch to remote work, maintaining their salaries, and finding ways to replace their regular social interactions. On the other hand, those who had a poor financial situation might have needed more emotional support. Socioeconomic status using different proxies has been previously linked to loneliness in the general population and among care partners (24, 45, 47).

We found an association between the changes in social isolation and overall and emotional loneliness, while the changes in social isolation were not statistically significantly associated with social loneliness. Previous studies have shown that socially isolated people can feel lonely, but not all lonely people are socially isolated (48). Theoretical models of loneliness and social exclusion have described the interplay between individuals’ needs, expectations, and their existing connections. These expectations stem from comparing one’s social connections with those of others (1, 46, 47). In a global context where social interactions were limited, emotional connections may have held greater significance than social contacts, potentially accounting for the absence of statistical association between social isolation and social loneliness. Interestingly, the dimension of physical loneliness has recently been brought to attention, considering that most restriction measures limited physical contact rather than social or emotional contact. In Germany, the first study about physical loneliness reported that its prevalence differed from the emotional and social dimensions of loneliness, and it increase during the first weeks of COVID-19 restrictions measures (49).

The present study has certain limitations. Firstly, this was a cross-sectional measure of the loneliness levels during the COVID-19 pandemic; therefore, no causal inferences can made. Secondly, the sample size for Latin American care partners might be biasing our estimations. We used bootstrap error to obtain more precise confidence intervals. Thirdly, only 10% of our sample comes from countries other than Mexico, Chile, and Brazil (see **Supplementary Table S1** published as **Supplementary Materials**), so the results might have external validity issues. Fourthly, the sample analysed in this study had mostly care partners with postgraduate education which is expected in online surveys because of computer access and the required skills to use the survey platforms. Thus, our sample is not necessarily representing the reality of all Latin American care partners and our results should be interpreted with caution. Fifthly, the respondents were volunteer participants who answered an online survey, which means selection bias might be present. Sixthly, pre-COVID-19 loneliness was retrospectively measured and was potentially affected by recall bias. To overcome this issue, we only modelled the during-COVID-19 measures, as they reflect the present of the care partners at the moment of the survey. Seventhly, physical restriction measures varied not only across countries but across time in the same country, therefore the impact might vary depending on where and when the survey was answered. Finally, despite the long list of variables explored, the explained variance for all the models was very low, which is an indication of unmeasured predictor factors and potential confounders. Additionally, the models for emotional and social isolation need to be taken as an exploratory attempt to describe the factors associated. We selected the best solution for both outcomes, but the models’ residuals were not normally distributed, which might indicate unmeasured variables better associated with emotional and social loneliness. There is a need for further exploration with a higher statistical power. Noteworthy, the model for overall loneliness showed an adequate goodness of fit. Finally, because of the nature of the survey, we were not able to calculate response rate and participation rates, and the missing data was up to 25%. We did not impute the data but, in order to improve the precision of the standard errors, bootstrap analyses using 1000 iterations with robust error were carried to calculate our regression models (50).

## Conclusion

To our knowledge, this is the first multinational study that evaluated Latin American care partners of persons with any enduring health condition, including physical and mental diseases. The results should be considered as an exploratory approach to describe the levels of overall, emotional, and social loneliness among family care partners pre and during the pandemic of COVID-19.

The COVID-19 pandemic, and the physical restrictions implemented, impacted global society at several levels to an extent that only time will tell. An increase in loneliness was a particularly relevant effect in vulnerable populations, such as care partners of people with long-term physical, cognitive, or mental health conditions.

The increase in the levels of social and emotional subtypes showed in our results should be considered when planning for public health interventions for mental health particularly for those with lower education and worse previous mental health. After the COVID-19 pandemic, a rise in mental health problems is expected and governments should focus their effects on at-risk populations like care partners.

The pre-pandemic levels of social and emotional loneliness were predictors of overall and specific loneliness during the pandemic. Future research should look for more evidence on risk factors for loneliness and its impact on care partners’ physical and mental health. In addition, longitudinal studies are required to provide more details about how loneliness impacts care partners and the general population.

## Data Availability

Restrictions apply to the availability of the CLIC data. To request data access, readers should contact the research leader Roger O'Sullivan (Roger.OSullivan@publichealth.ie).

## References

[1] Burholt V Winter B Aartsen M Constantinou C Dahlberg L Feliciano V. A critical review and development of a conceptual model of exclusion from social relations for older people. Eur J Ageing. (2020) 17:3–19. doi: 10.1007/s10433-019-00506-0 32158368 PMC7040153

[2] de Jong-Gierveld J. Developing and testing a model of loneliness. J Pers Soc Psychol. (1987) 53:119–28. doi: 10.1037//0022-3514.53.1.119 3612484

[3] Weiss RS. Loneliness: The experience of emotional and social isolation. Cambridge, Massachusetts. U.S.A.: The MIT Press (1973).

[4] Cacioppo S Grippo AJ London S Goossens L Cacioppo JT. Loneliness: clinical import and interventions. Perspect Psychol Sci. (2015) 10:238–49. doi: 10.1177/1745691615570616 PMC4391342 25866548

[5] De Jong Gierveld J Van Tilburg T. The De Jong Gierveld short scales for emotional and social loneliness: tested on data from 7 countries in the UN generations and gender surveys. Eur J Ageing. (2010) 7:121–30. doi: 10.1007/s10433-010-0144-6 PMC2921057 20730083

[6] Gierveld J. A review of loneliness: concept and definitions, determinants and consequences. Rev Clin Gerontology. (1998) 8:73–80. doi: 10.1017/S0959259898008090

[7] Gierveld J Tilburg T Dykstra PA. New ways of theorizing and conducting research in the field of loneliness and social isolation. In: Vangelisti AL Perlman D, editors. The Cambridge Handbook of Personal Relationships, 2 ed. Cambridge, U.K.: Cambridge University Press (2018). p. 391–404. doi: 10.1017/9781316417867.031

[8] Gierveld JDJ Tilburg TV. A 6-item scale for overall, emotional, and social loneliness: confirmatory tests on survey data. Res Aging. (2006) 28:582–98. doi: 10.1177/0164027506289723

[9] Perlman D Peplau LA. Toward a social psychology of loneliness. Pers Relat. (1981) 3:31–56.

[10] Dahlberg L McKee KJ Frank A Naseer M. A systematic review of longitudinal risk factors for loneliness in older adults. Aging Ment Health. (2022) 26:225–49. doi: 10.1080/13607863.2021.1876638 33563024

[11] Hawkley LC Capitanio JP. Perceived social isolation, evolutionary fitness and health outcomes: a lifespan approach. Philos Trans R Soc Lond B Biol Sci. (2015) 370. doi: 10.1098/rstb.2014.0114 PMC4410380 25870400

[12] Holt-Lunstad J Smith TB Baker M Harris T Stephenson D. Loneliness and social isolation as risk factors for mortality: a meta-analytic review. Perspect Psychol Sci. (2015) 10:227–37. doi: 10.1177/1745691614568352 25910392

[13] Vindegaard N Benros ME. COVID-19 pandemic and mental health consequences: Systematic review of the current evidence. Brain Behav Immun. (2020) 89:531–42. doi: 10.1016/j.bbi.2020.05.048 PMC7260522 32485289

[14] Allel K Tapia-Muñoz T Morris W. Country-level factors associated with the early spread of COVID-19 cases at 5, 10 and 15 days since the onset. Glob Public Health. (2020) 15:1589–602. doi: 10.1080/17441692.2020.1814835 32894686

[15] O'Sullivan R Burns A Leavey G Leroi I Burholt V Lubben J. Impact of the COVID-19 pandemic on loneliness and social isolation: A multi-country study. Int J Environ Res Public Health. (2021) 18:9982. doi: 10.3390/ijerph18199982 34639283 PMC8508181

[16] Bu F Steptoe A Fancourt D. Loneliness during a strict lockdown: Trajectories and predictors during the COVID-19 pandemic in 38,217 United Kingdom adults. Soc Sci Med. (2020) 265:113521. doi: 10.1016/j.socscimed.2020.113521 33257177 PMC7768183

[17] Bu F Steptoe A Fancourt D. Who is lonely in lockdown? Cross-cohort analyses of predictors of loneliness before and during the COVID-19 pandemic. Public Health. (2020) 186:31–4. doi: 10.1016/j.puhe.2020.06.036 32768621 PMC7405905

[18] O’Shea BQ Finlay JM Kler J Joseph CA Kobayashi LC. Loneliness among US adults aged ≥55 early in the COVID-19 pandemic: findings from the COVID-19 coping study. Public Health Rep. (2021) 136:754–64. doi: 10.1177/00333549211029965 PMC8579390 34283657

[19] Peng S Roth AR. Social isolation and loneliness before and during the COVID-19 pandemic: A longitudinal study of U.S. Adults older than 50. Journals Gerontology: Ser B. (2021) 77:gbab068. doi: 10.1093/geronb/gbab068 PMC8083229 33870414

[20] Khan MSR Kadoya Y. Loneliness during the COVID-19 pandemic: A comparison between older and younger people. Int J Environ Res Public Health. (2021) 18:7871. doi: 10.3390/ijerph18157871 34360164 PMC8345648

[21] Bramboeck V Moeller K Marksteiner J Kaufmann L. Loneliness and burden perceived by family caregivers of patients with Alzheimer disease. Am J Alzheimer's Dis Other Dementias®. (2020) 35:1533317520917788. doi: 10.1177/1533317520917788 PMC10624003 32281389

[22] Carbone EA de Filippis R Roberti R Rania M Destefano L Russo E. The mental health of caregivers and their patients with dementia during the COVID-19 pandemic: A systematic review. Front Psychol. (2021) 12:782833. doi: 10.3389/fpsyg.2021.782833 35002872 PMC8740146

[23] Hajek A Kretzler B König HH. Informal caregiving, loneliness and social isolation: A systematic review. Int J Environ Res Public Health. (2021) 18. doi: 10.3390/ijerph182212101 PMC8618455 34831857

[24] Victor C Rippon I Quinn C Nelis SM Martyr A Hart N. The prevalence and predictors of loneliness in caregivers of people with dementia: findings from the IDEAL programme. Aging Ment Health. (2021) 25:1232–8. doi: 10.1080/13607863.2020.1753014 32306759

[25] Grycuk E Chen Y Almirall-Sanchez A Higgins D Galvin M Kane J. Care burden, loneliness, and social isolation in caregivers of people with physical and brain health conditions in English-speaking regions: Before and during the COVID-19 pandemic. Int J Geriatric Psychiatry. (2022) 37. doi: 10.1002/gps.5734 PMC9324775 35574817

[26] Wormald A McGlinchey E D'Eath M Leroi I Lawlor B McCallion P. Impact of COVID-19 pandemic on caregivers of people with an intellectual disability, in comparison to carers of those with other disabilities and with mental health issues: A multicountry study. Int J Environ Res Public Health. (2023) 20. doi: 10.3390/ijerph20043256 PMC9965347 36833954

[27] Bristol AA Mata AC Mickens M Dassel KB Ellington L Scammon D. You feel very isolated”: effects of COVID-19 pandemic on caregiver social connections. Gerontology Geriatric Med. (2021) 7:23337214211060166. doi: 10.1177/23337214211060166 PMC8724994 34993276

[28] The Lancet. COVID-19 in Latin America-emergency and opportunity. Lancet. (2021) 398:93. doi: 10.1016/s0140-6736(21)01551-8 34246349 PMC8266269

[29] Ibáñez A Pina-Escudero SD Possin KL Quiroz YT Peres FA Slachevsky A. Dementia caregiving across Latin America and the Caribbean and brain health diplomacy. Lancet Healthy Longevity. (2021) 2:e222–31. doi: 10.1016/S2666-7568(21)00031-3 PMC8594860 34790905

[30] Russell DW. UCLA Loneliness Scale (Version 3): reliability, validity, and factor structure. J Pers Assess. (1996) 66:20–40. doi: 10.1207/s15327752jpa6601_2 8576833

[31] Tapia-Munoz T Ajnakina O Fancourt D Steptoe A. Personality traits and loneliness among older people in the UK: Cross-sectional and longitudinal analysis from the English Longitudinal Study of Ageing. Eur J Pers. (2024) 38:599–614. doi: 10.1177/08902070231206196

[32] Bu F Steptoe A Fancourt D. Who is lonely in lockdown? Cross-cohort analyses of predictors of loneliness before and during the COVID-19 pandemic. Public Health. (2020) 186:31–4. doi: 10.1016/j.puhe.2020.06.036 PMC7405905 32768621

[33] Lee SL Pearce E Ajnakina O Johnson S Lewis G Mann F. The association between loneliness and depressive symptoms among adults aged 50 years and older: a 12-year population-based cohort study. Lancet Psychiatry. (2021) 8:48–57. doi: 10.1016/S2215-0366(20)30383-7 33181096 PMC8009277

[34] Maharani A Zaidi SNZ Jury F Vatter S Hill D Leroi I. The long-term impact of loneliness and social isolation on depression and anxiety in memory clinic attendees and their care partners: A longitudinal actor-partner interdependence model. Alzheimers Dement (N Y). (2022) 8:e12235. doi: 10.1002/trc2.12235 35505900 PMC9053374

[35] Kola L Kumar M Kohrt BA Fatodu T Olayemi BA Adefolarin AO. Strengthening public mental health during and after the acute phase of the COVID-19 pandemic. Lancet. (2022) 399:1851–2. doi: 10.1016/S0140-6736(22)00523-2 PMC8947778 35339230

[36] Victor C Pikhartova J. Lonely places or lonely people? Investigating the relationship between loneliness and place of residence. BMC Public Health. (2020) 20:778. doi: 10.1186/s12889-020-08703-8 32456626 PMC7251825

[37] Vasileiou K Barnett J Barreto M Vines J Atkinson M Lawson S. Experiences of loneliness associated with being an informal caregiver: A qualitative investigation. Front Psychol. (2017) 8:585. doi: 10.3389/fpsyg.2017.00585 28469589 PMC5395647

[38] Parker LJ Fabius C Rivers E Taylor JL. Is dementia-specific caregiving compared with non-dementia caregiving associated with physical difficulty among caregivers for community-dwelling adults? J Appl Gerontol. (2022) 41:1074–80. doi: 10.1177/07334648211014352 PMC8664093 34041929

[39] Dahlberg L Andersson L McKee KJ Lennartsson C. Predictors of loneliness among older women and men in Sweden: A national longitudinal study. Aging Ment Health. (2015) 19:409–17. doi: 10.1080/13607863.2014.944091 25126996

[40] de Jong Gierveld J Dykstra PA Schenk N. Living arrangements, intergenerational support types and older adult loneliness in Eastern and Western Europe. Demographic Res. (2012) S11:167–200. doi: 10.4054/DemRes.2012.27.7

[41] Abdellaoui A Chen HY Willemsen G Ehli EA Davies GE Verweij KJH. Associations between loneliness and personality are mostly driven by a genetic association with Neuroticism. J Pers. (2019) 87:386–97. doi: 10.1111/jopy.12397 PMC6231981 29752830

[42] Abdellaoui A Nivard MG Hottenga JJ Fedko I Verweij KJH Baselmans BML. Predicting loneliness with polygenic scores of social, psychological and psychiatric traits. Genes Brain Behav. (2018) 17:e12472. doi: 10.1111/gbb.12472 29573219 PMC6464630

[43] Sung P May-Ling Lee J Chan A. Lonely in a crowd: social isolation profiles and caregiver burden among family caregivers of community-dwelling older adults with cognitive impairment. J Aging Health. (2023) 35:419–29. doi: 10.1177/08982643221137939 36330754

[44] Bosma H Jansen M Schefman S Hajema KJ Feron F. Lonely at the bottom: a cross-sectional study on being ill, poor, and lonely. Public Health. (2015) 129:185–7. doi: 10.1016/j.puhe.2014.11.016 25682907

[45] Algren MH Ekholm O Nielsen L Ersbøll AK Bak CK Andersen PT. Social isolation, loneliness, socioeconomic status, and health-risk behaviour in deprived neighbourhoods in Denmark: A cross-sectional study. SSM Popul Health. (2020) 10:100546. doi: 10.1016/j.ssmph.2020.100546 32042889 PMC6997896

[46] de Jong Gierveld J Tesch-Römer C. Loneliness in old age in Eastern and Western European societies: theoretical perspectives. Eur J Ageing. (2012) 9:285–95. doi: 10.1007/s10433-012-0248-2 PMC5549113 28804428

[47] Aartsen M Morgan D Dahlberg L Waldegrave C Mikulionienė S Rapolienė G. Exclusion from social relations and loneliness: individual and country-level changes. Innovation Aging. (2020) 4:712–3. doi: 10.1093/geroni/igaa057.2509

[48] NatCen Social Research. User Guide to the Main Interview Datasets: waves 1 to 9. (London, UK: UK Data Service) (2020).

[49] Landmann H Rohmann A. When loneliness dimensions drift apart: Emotional, social and physical loneliness during the COVID-19 lockdown and its associations with age, personality, stress and well-being. Int J Psychol. (2022) 57:63–72. doi: 10.1002/ijop.12772 33973238 PMC8239761

[50] Efron B. Missing data, imputation, and the bootstrap. J Am Stat Assoc. (1994) 89:463–75. doi: 10.2307/2290846

