# Supplementary material for: Factors associated with loneliness in Latin-American family care partners during the COVID-19 pandemic
Source: Front Psychiatry. 2024 Nov 12;15:1286141. doi: 10.3389/fpsyt.2024.1286141 (PMC11588483; doi:10.3389/fpsyt.2024.1286141)
Supplement: Supplementary file 1 [file DataSheet1.docx]

**SUPPLEMENTARY MATERIALS**

**“Factors associated with overall, emotional, and social loneliness in Latin-American family care partners during the COVID-19 pandemic”**

**Section A. Descriptive analysis**

Table S1: participant’s countries

Figure S1. Distribution for total, emotional and social loneliness at baselines and follow-up.

Figure S2. Overall loneliness by age and gender (unadjusted mean level of loneliness.

**Section B. Unadjusted models for loneliness**

Table S2. Single regression models for overall loneliness

Table S3. Single regression models for emotional loneliness

Table S4. Single regression models for social loneliness

**Section C. Models fit using residual distributions**

Figure S3. Residual distribution for overall, emotional, and social loneliness

**Section A. Descriptive analysis**

Table S1: participant’s countries

|  | Freq. | Percent |
| --- | --- | --- |
| Antigua/Barbuda | 0.41 | 0.41 |
| Argentina | 1 | 0.41 |
| Brazil | 45 | 18.29 |
| Chile | 63 | 25.61 |
| Costa Rica | 1 | 0.41 |
| Guatemala | 2 | 0.81 |
| Mexico | 113 | 45.93 |
| Panama | 10 | 4.07 |
| Peru | 8 | 3.25 |
| Saint Lucia | 1 | 0.41 |
| Venezuela | 1 | 0.41 |
| Total | 246 | 100 |

Figure S1. Distribution for total, emotional and social loneliness at baselines and follow-up

| **Total Loneliness** | |
| --- | --- |
| 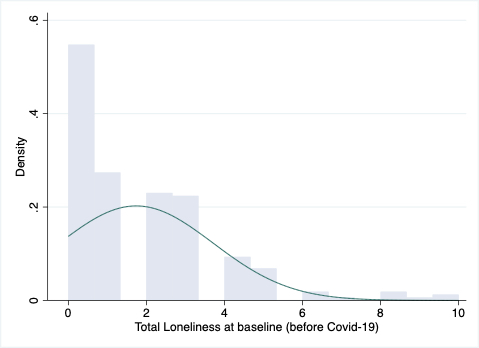 | 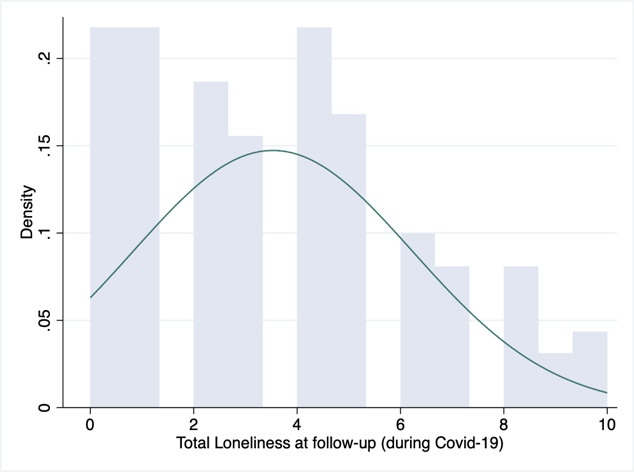 |
| **Emotional Loneliness** | |
| 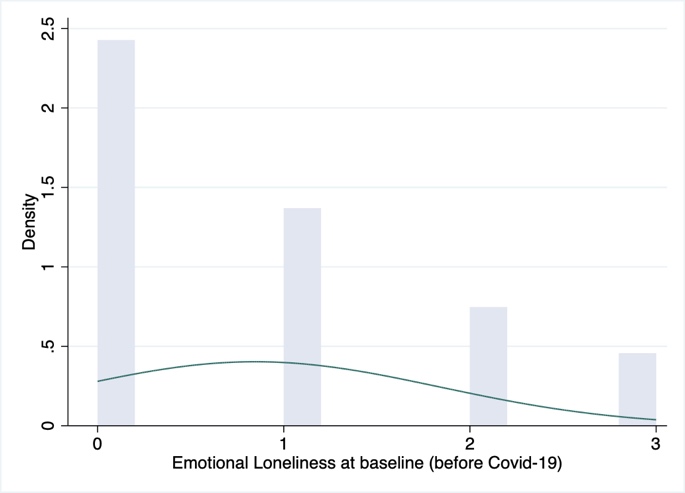 | 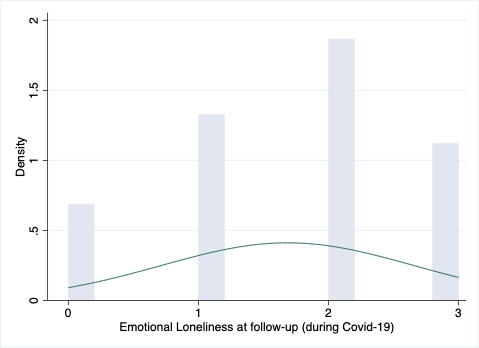 |
| **Social Loneliness** | |
| 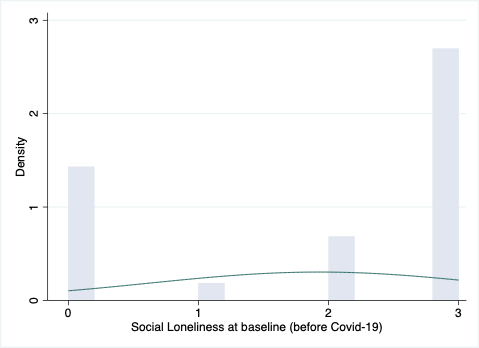 | 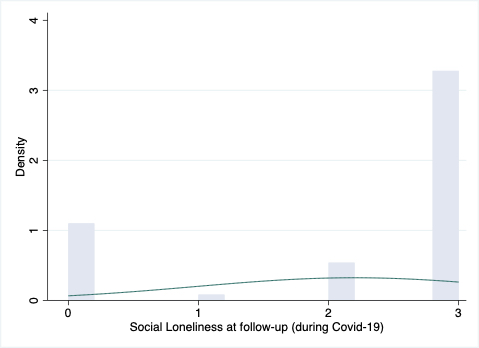 |
|  |  |

Figure S2. overall loneliness by age and gender (unadjusted mean level of loneliness)


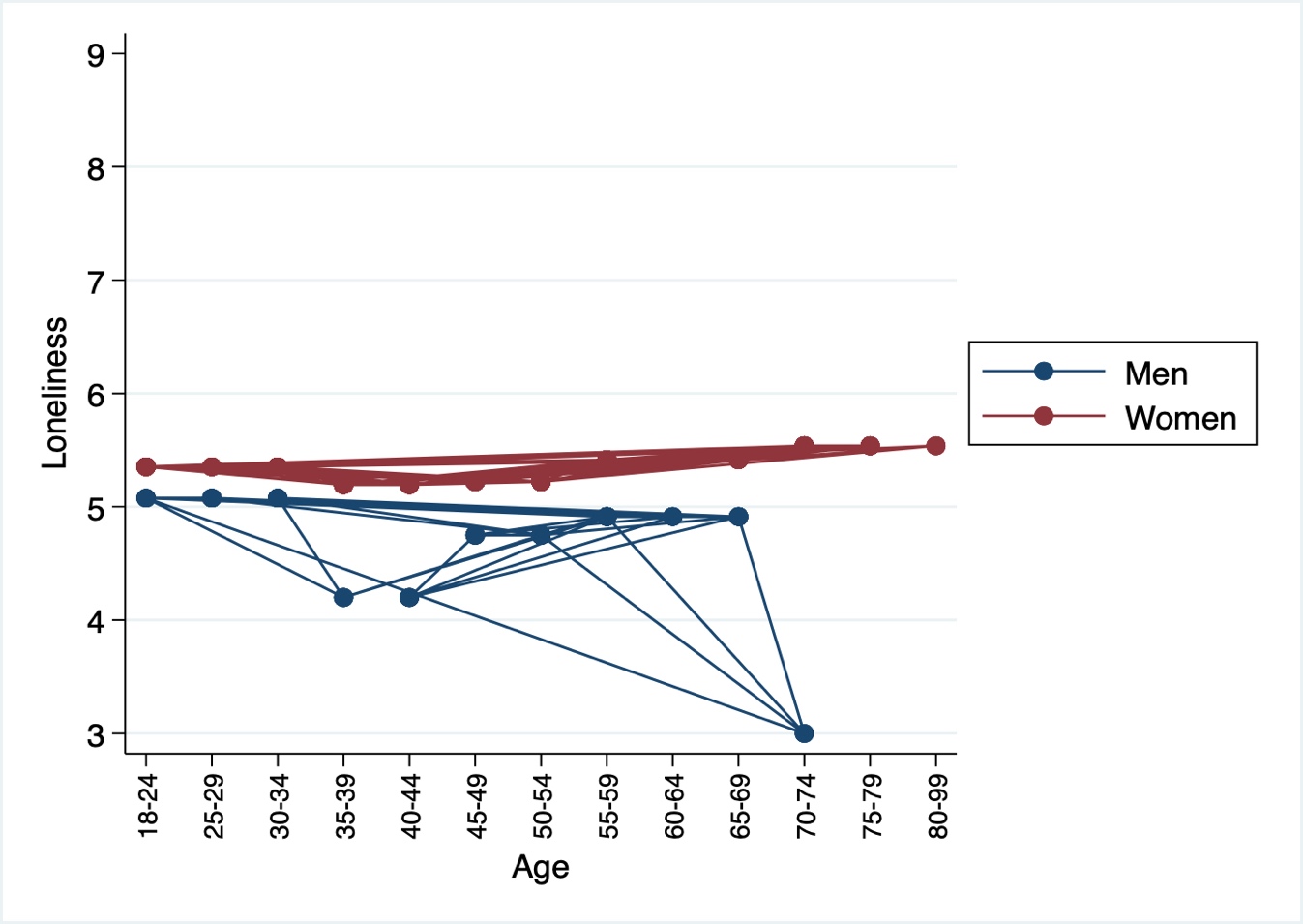


**Section B. Unadjusted models for loneliness**

**Table S2. Single regression models for overall loneliness (n=246)**

|  | Coefficient | SE | t | p-value | 95% CI | |
| --- | --- | --- | --- | --- | --- | --- |
| Educational level^a^ | 0.493 | 0.237 | 2.08 | 0.037 | 0.029 | 0.957 |
| Constant | 4.956 | 0.142 | 34.92 | 0.000 | 4.678 | 5.234 |
|  | Coefficient | SE | t | p-value | 95% CI | |
| Gender^b^ | 0.514 | 0.277 | 1.86 | 0.063 | -0.028 | 1.056 |
| Constant | 4.769 | 0.245 | 19.5 | 0.000 | 4.290 | 5.249 |
|  | Coefficient | SE | t | p-value | 95% CI | |
| Age^c^ | 0.321 | 0.226 | 1.42 | 0.156 | -0.122 | 0.764 |
| Constant | 5.000 | 0.166 | 30.13 | 0.000 | 4.675 | 5.325 |
|  | Coefficient | SE | t | p-value | 95% CI | |
| Kin relationship^d^ | -0.794 | 0.369 | -2.15 | 0.032 | -1.517 | -0.070 |
| Constant | 6.678 | 0.713 | 9.37 | 0.000 | 5.282 | 8.075 |
|  | Coefficient | SE | t | p-value | 95% CI | |
| Marital status^e^ | -0.214 | 0.246 | -0.87 | 0.385 | -0.695 | 0.268 |
| Constant | 5.298 | 0.195 | 27.22 | 0.000 | 4.917 | 5.680 |
|  | Coefficient | SE | t | p-value | 95% CI | |
| Living arrangements^f^ | -0.009 | 0.573 | -0.01 | 0.988 | -1.132 | 1.115 |
| Constant | 5.175 | 0.118 | 43.96 | 0.000 | 4.944 | 5.406 |
|  | Coefficient | SE | t | p-value | 95% CI |  |
| Physical health binary^g^ | 0.347 | 0.286 | 1.21 | 0.226 | -0.215 | 0.908 |
| Constant | 5.110 | 0.127 | 40.25 | 0.000 | 4.861 | 5.359 |
|  | Coefficient | SE | t | p-value | 95% CI |  |
| Mental health binary^g^ | 1.064 | 0.348 | 3.06 | 0.002 | 0.383 | 1.745 |
| Constant | 5.019 | 0.122 | 41.19 | 0.000 | 4.780 | 5.258 |
|  | Coefficient | SE | t | p-value | 95% CI |  |
| Burden^h^ | 1.072 | 0.241 | 4.45 | 0.000 | 0.600 | 1.544 |
| Constant | 4.800 | 0.124 | 38.78 | 0.000 | 4.557 | 5.043 |
|  | Coefficient | SE | t | p-value | 95% CI |  |
| Finances met needs^i^ | 0.210 | 0.162 | 1.3 | 0.194 | -0.107 | 0.527 |
| Constant | 4.779 | 0.330 | 14.49 | 0.000 | 4.132 | 5.425 |
|  | Coefficient | SE | t | p-value | 95% CI |  |
| Covid hospitalization^j^ | 0.084 | 0.139 | 0.61 | 0.545 | -0.189 | 0.357 |
| Constant | 4.870 | 0.510 | 9.54 | 0.000 | 3.870 | 5.870 |
|  | Coefficient | SE | t | p-value | 95% CI |  |
| Anyone died^k^ | 0.071 | 0.238 | 0.3 | 0.767 | -0.397 | 0.538 |
| Constant | 5.152 | 0.139 | 36.97 | 0.000 | 4.878 | 5.425 |
|  | Coefficient | SE | t | p-value | 95% CI |  |
| Covid death^k^ | 0.257 | 0.297 | 0.86 | 0.388 | -0.326 | 0.840 |
| Constant | 5.007 | 0.145 | 34.49 | 0.000 | 4.723 | 5.292 |
|  | Coefficient | SE | t | p-value | 95% CI |  |
| Alcohol consumption | -0.054 | 0.108 | -0.5 | 0.620 | -0.265 | 0.158 |
| Constant | 5.338 | 0.351 | 15.2 | 0.000 | 4.650 | 6.027 |
|  | Coefficient | SE | t | p-value | 95% CI |  |
| Smoking | 0.016 | 0.195 | 0.08 | 0.933 | -0.365 | 0.398 |
| Constant | 5.122 | 0.746 | 6.87 | 0.000 | 3.660 | 6.584 |
| Sleeping^l^ | Coefficient | SE | t | p-value | 95% CI |  |
| More | 1.252 | 0.430 | 2.91 | 0.004 | 0.409 | 2.095 |
| About the same | 0.292 | 0.416 | 0.7 | 0.482 | -0.523 | 1.107 |
| Constant | 4.438 | 0.384 | 11.56 | 0.000 | 3.685 | 5.190 |
|  | Coefficient | SE | t | p-value | 95% CI |  |
| Worse social isolation^m^ | 0.974 | 0.267 | 3.65 | 0.000 | 0.451 | 1.498 |
| Constant | 4.941 | 0.123 | 40.25 | 0.000 | 4.701 | 5.182 |
|  | Coefficient | SE | t | p-value | 95% CI | |
| Dementia carer^m^ | -0.161 | 0.272 | -0.59 | 0.555 | -0.695 | 0.373 |
| Constant | 5.234 | 0.139 | 37.62 | 0.000 | 4.962 | 5.507 |
| Diagnosis^n^ | Coefficient | SE | t | p-value | 95% CI | |
| Physical | 0.113 | 0.341 | 0.330 | 0.741 | -0.555 | 0.781 |
| Mental | 0.272 | 0.433 | 0.630 | 0.530 | -0.577 | 1.122 |
| Intellectual | 0.492 | 0.615 | 0.800 | 0.424 | -0.714 | 1.697 |
| Others | 0.330 | 0.357 | 0.930 | 0.354 | -0.369 | 1.029 |
| Dual | 0.687 | 0.479 | 1.430 | 0.151 | -0.251 | 1.626 |
| Constant | 4.963 | 0.260 | 19.080 | 0.000 | 4.453 | 5.473 |
|  | Coefficient | SE | t | p-value | 95% CI | |
| Children^m^ | -0.289 | 0.240 | -1.200 | 0.229 | -0.759 | 0.182 |
| Constant | 5.374 | 0.194 | 27.730 | 0.000 | 4.994 | 5.753 |

**Notes.***p-value<0.05 **p-value<0.01 ***p-value<0.001. ^a^ref cat “postgraduate ^b^ref cat “less than 50 years old” ^c^ref cat “men” ^d^ref cat “family member” ^e^ref cat “married or in a partnership” ^f^ref cat “living alone” ^g^ref cat “poor/fair mental health” ^h^ref cat “no burden” ^i^ref cat “very well” ^j^ref cat “someone close to me” ^k^ref cat “no” ^l^ref cat “less than usual” ^m^ref cat “yes” ^n^ref cat ”dementia”. 95% CI first column depicts min value; second column depicts max value.

**Table S3. Single regression models for emotional loneliness (n=246)**

|  | Coefficient | SE | t | p-value | 95% CI | |
| --- | --- | --- | --- | --- | --- | --- |
| Educational level^a^ | 0.185 | 0.125 | 1.470 | 0.142 | -0.062 | 0.432 |
| Constant | 1.392 | 0.192 | 7.270 | 0.000 | 1.015 | 1.769 |
|  | Coefficient | SE | t | p-value | 95% CI | |
| Gender^b^ | 0.250 | 0.153 | 1.640 | 0.103 | -0.051 | 0.550 |
| Constant | 1.462 | 0.135 | 10.790 | 0.000 | 1.195 | 1.728 |
|  | Coefficient | SE | t | p-value | 95% CI | |
| Age^c^ | 0.062 | 0.126 | 0.490 | 0.625 | -0.186 | 0.309 |
| Constant | 1.625 | 0.093 | 17.520 | 0.000 | 1.442 | 1.808 |
|  | Coefficient | SE | t | p-value | 95% CI | |
| Kin relationship^d^ | -0.333 | 0.174 | -1.920 | 0.057 | -0.675 | 0.009 |
| Constant | 2.298 | 0.339 | 6.770 | 0.000 | 1.629 | 2.967 |
|  | Coefficient | SE | t | p-value | 95% CI | |
| Marital status^e^ | 0.025 | 0.127 | 0.200 | 0.845 | -0.225 | 0.274 |
| Constant | 1.644 | 0.096 | 17.080 | 0.000 | 1.455 | 1.834 |
|  | Coefficient | SE | t | p-value | 95% CI | |
| Living arrangements^f^ | -0.429 | 0.289 | -1.480 | 0.139 | -0.999 | 0.140 |
| Constant | 1.679 | 0.064 | 26.280 | 0.000 | 1.554 | 1.805 |
|  | Coefficient | SE | t | p-value | 95% CI | |
| Physical health binary^g^ | 0.340 | 0.159 | 2.140 | 0.034 | 0.026 | 0.653 |
| Constant | 1.595 | 0.069 | 23.180 | 0.000 | 1.459 | 1.731 |
|  | Coefficient | SE | t | p-value | 95% CI | |
| Mental health binary^g^ | 0.660 | 0.172 | 3.840 | 0.000 | 0.321 | 0.999 |
| Constant | 1.562 | 0.066 | 23.730 | 0.000 | 1.432 | 1.692 |
|  | Coefficient | SE | t | p-value | 95% CI | |
| Burden^h^ | 0.293 | 0.130 | 2.250 | 0.025 | 0.037 | 0.549 |
| Constant | 1.556 | 0.077 | 20.250 | 0.000 | 1.405 | 1.708 |
|  | Coefficient | SE | t | p-value | 95% CI | |
| Finances met needs^i^ | 0.207 | 0.084 | 2.450 | 0.015 | 0.041 | 0.372 |
| Constant | 1.269 | 0.170 | 7.450 | 0.000 | 0.933 | 1.605 |
|  | Coefficient | SE | t | p-value | 95% CI | |
| Covid hospitalization^j^ | 0.054 | 0.077 | 0.700 | 0.486 | -0.098 | 0.205 |
| Constant | 1.470 | 0.281 | 5.220 | 0.000 | 0.916 | 2.024 |
|  | Coefficient | SE | t | p-value | 95% CI | |
| Anyone died^k^ | -0.006 | 0.133 | -0.050 | 0.962 | -0.269 | 0.256 |
| Constant | 1.661 | 0.076 | 21.720 | 0.000 | 1.510 | 1.811 |
|  | Coefficient | SE | t | p-value | 95% CI | |
| Covid death^k^ | 0.153 | 0.159 | 0.960 | 0.337 | -0.160 | 0.467 |
| Constant | 1.583 | 0.083 | 18.960 | 0.000 | 1.418 | 1.747 |
|  | Coefficient | SE | t | p-value | 95% CI | |
| Alcohol consumption | 0.045 | 0.056 | 0.800 | 0.424 | -0.065 | 0.155 |
| Constant | 1.521 | 0.182 | 8.350 | 0.000 | 1.163 | 1.880 |
|  | Coefficient | SE | t | p-value | 95% CI | |
| Smoking | -0.059 | 0.101 | -0.590 | 0.558 | -0.258 | 0.139 |
| Constant | 1.885 | 0.387 | 4.870 | 0.000 | 1.123 | 2.648 |
| Sleeping^l^ | Coefficient | SE | t | p-value | 95% CI | |
| More | 0.629 | 0.252 | 2.500 | 0.013 | 0.133 | 1.124 |
| About the same | 0.093 | 0.253 | 0.370 | 0.713 | -0.405 | 0.590 |
| Constant | 1.313 | 0.236 | 5.560 | 0.000 | 0.847 | 1.778 |
|  | Coefficient | SE | t | p-value | 95% CI | |
| Worse social isolation^m^ | 0.583 | 0.142 | 4.110 | 0.000 | 0.304 | 0.862 |
| Constant | 1.519 | 0.069 | 21.870 | 0.000 | 1.382 | 1.656 |
|  | Coefficient | SE | t | p-value | 95% CI | |
| Dementia carer^m^ | -0.068 | 0.141 | -0.480 | 0.629 | -0.345 | 0.209 |
| Constant | 1.686 | 0.074 | 22.640 | 0.000 | 1.539 | 1.832 |
| Diagnosis^n^ | Coefficient | SE | t | p-value | 95% CI | |
| Physical | -0.072 | 0.188 | -0.390 | 0.700 | -0.440 | 0.296 |
| Mental | -0.060 | 0.210 | -0.290 | 0.775 | -0.472 | 0.352 |
| Intellectual | 0.443 | 0.314 | 1.410 | 0.159 | -0.173 | 1.059 |
| Others | 0.128 | 0.173 | 0.740 | 0.459 | -0.211 | 0.466 |
| Dual | -0.048 | 0.235 | -0.200 | 0.838 | -0.509 | 0.412 |
| Constant | 1.648 | 0.130 | 12.690 | 0.000 | 1.394 | 1.903 |
|  | Coefficient | SE | t | p-value | 95% CI | |
| Children^m^ | -0.102 | 0.129 | -0.790 | 0.428 | -0.356 | 0.151 |
| Constant | 1.736 | 0.102 | 17.020 | 0.000 | 1.535 | 1.937 |

**Notes.***p-value<0.05 **p-value<0.01 ***p-value<0.001. ^a^ref cat “postgraduate ^b^ref cat “less than 50 years old” ^c^ref cat “men” ^d^ref cat “family member” ^e^ref cat “married or in a partnership” ^f^ref cat “living alone” ^g^ref cat “poor/fair mental health” ^h^ref cat “no burden” ^i^ref cat “very well” ^j^ref cat “someone close to me” ^k^ref cat “no” ^l^ref cat “less than usual” ^m^ref cat “yes”. ^n^ref cat ”dementia”. 95% CI first column depicts min value; second column depicts max value.

**Table S4. Single regression models for social loneliness (n=246)**

|  | Coefficient | SE | t | p-value | 95% CI | |
| --- | --- | --- | --- | --- | --- | --- |
| Educational level^a^ | 0.508 | 0.155 | 3.280 | 0.001 | 0.203 | 0.814 |
| Constant | 1.470 | 0.237 | 6.210 | 0.000 | 1.004 | 1.936 |
|  | Coefficient | SE | t | p-value | 95% CI | |
| Gender^b^ | 0.185 | 0.192 | 0.960 | 0.338 | -0.194 | 0.564 |
| Constant | 2.058 | 0.171 | 12.040 | 0.000 | 1.721 | 2.394 |
|  | Coefficient | SE | t | p-value | 95% CI | |
| Age^c^ | -0.250 | 0.157 | -1.590 | 0.114 | -0.559 | 0.060 |
| Constant | 2.339 | 0.116 | 20.160 | 0.000 | 2.111 | 2.568 |
|  | Coefficient | SE | t | p-value | 95% CI | |
| Kin relationship^d^ | 0.154 | 0.220 | 0.700 | 0.483 | -0.278 | 0.587 |
| Constant | 1.907 | 0.430 | 4.440 | 0.000 | 1.061 | 2.753 |
|  | Coefficient | SE | t | p-value | 95% CI | |
| Marital status^e^ | 0.119 | 0.159 | 0.750 | 0.456 | -0.195 | 0.432 |
| Constant | 2.135 | 0.121 | 17.650 | 0.000 | 1.896 | 2.373 |
|  | Coefficient | SE | t | p-value | 95% CI | |
| Living arrangements^f^ | -0.126 | 0.365 | -0.350 | 0.730 | -0.846 | 0.593 |
| Constant | 2.209 | 0.081 | 27.380 | 0.000 | 2.050 | 2.368 |
|  | Coefficient | SE | t | p-value | 95% CI | |
| Physical health binary^g^ | 0.071 | 0.202 | 0.350 | 0.726 | -0.327 | 0.468 |
| Constant | 2.190 | 0.087 | 25.100 | 0.000 | 2.018 | 2.362 |
|  | Coefficient | SE | t | p-value | 95% CI | |
| Mental health binary^g^ | 0.478 | 0.221 | 2.170 | 0.031 | 0.043 | 0.912 |
| Constant | 2.133 | 0.084 | 25.280 | 0.000 | 1.967 | 2.300 |
|  | Coefficient | SE | t | p-value | 95% CI | |
| Burden^h^ | 0.224 | 0.164 | 1.360 | 0.175 | -0.100 | 0.548 |
| Constant | 2.125 | 0.097 | 21.860 | 0.000 | 1.933 | 2.317 |
|  | Coefficient | SE | t | p-value | 95% CI | |
| Finances met needs^i^ | 0.163 | 0.107 | 1.530 | 0.127 | -0.047 | 0.373 |
| Constant | 1.895 | 0.216 | 8.780 | 0.000 | 1.470 | 2.320 |
|  | Coefficient | SE | t | p-value | 95% CI | |
| Covid hospitalization^j^ | -0.102 | 0.096 | -1.060 | 0.289 | -0.292 | 0.088 |
| Constant | 2.570 | 0.354 | 7.270 | 0.000 | 1.874 | 3.267 |
|  | Coefficient | SE | t | p-value | 95% CI | |
| Anyone died^k^ | 0.341 | 0.166 | 2.050 | 0.041 | 0.014 | 0.668 |
| Constant | 2.091 | 0.095 | 21.940 | 0.000 | 1.903 | 2.279 |
|  | Coefficient | SE | t | p-value | 95% CI | |
| Covid death^k^ | 0.084 | 0.201 | 0.420 | 0.676 | -0.312 | 0.481 |
| Constant | 2.180 | 0.106 | 20.630 | 0.000 | 1.971 | 2.388 |
|  | Coefficient | SE | t | p-value | 95% CI | |
| Alcohol consumption | -0.071 | 0.070 | -1.010 | 0.313 | -0.210 | 0.067 |
| Constant | 2.421 | 0.229 | 10.580 | 0.000 | 1.970 | 2.871 |
|  | Coefficient | SE | t | p-value | 95% CI | |
| Smoking | -0.185 | 0.126 | -1.470 | 0.143 | -0.434 | 0.063 |
| Constant | 2.902 | 0.485 | 5.990 | 0.000 | 1.947 | 3.857 |
| Sleeping^l^ | Coefficient | SE | t | p-value | 95% CI | |
| More | -0.027 | 0.328 | -0.080 | 0.935 | -0.674 | 0.620 |
| About the same | -0.213 | 0.330 | -0.650 | 0.518 | -0.863 | 0.436 |
| Constant | 2.313 | 0.308 | 7.500 | 0.000 | 1.705 | 2.920 |
|  | Coefficient | SE | t | p-value | 95% CI | |
| Worse social isolation^m^ | 0.335 | 0.183 | 1.830 | 0.069 | -0.026 | 0.695 |
| Constant | 2.123 | 0.090 | 23.680 | 0.000 | 1.946 | 2.300 |
|  | Coefficient | SE | t | p-value | 95% CI | |
| Dementia carer^m^ | -0.204 | 0.176 | -1.160 | 0.246 | -0.550 | 0.142 |
| Constant | 2.263 | 0.093 | 24.370 | 0.000 | 2.080 | 2.446 |
| Diagnosis^n^ | Coefficient | SE | t | p-value | 95% CI | |
| Physical | 0.231 | 0.231 | 1.000 | 0.318 | -0.222 | 0.683 |
| Mental | 0.401 | 0.277 | 1.450 | 0.148 | -0.142 | 0.944 |
| Intellectual | 0.200 | 0.478 | 0.420 | 0.675 | -0.736 | 1.137 |
| Others | 0.260 | 0.248 | 1.050 | 0.294 | -0.225 | 0.745 |
| Dual | 0.419 | 0.324 | 1.290 | 0.196 | -0.216 | 1.053 |
| Constant | 1.981 | 0.183 | 10.830 | 0.000 | 1.623 | 2.340 |
|  | Coefficient | SE | t | p-value | 95% CI | |
| Children^m^ | -0.140 | 0.163 | -0.860 | 0.391 | -0.460 | 0.181 |
| Constant | 2.297 | 0.129 | 17.830 | 0.000 | 2.043 | 2.550 |

**Notes.***p-value<0.05 **p-value<0.01 ***p-value<0.001. ^a^ref cat “postgraduate ^b^ref cat “less than 50 years old” ^c^ref cat “men” ^d^ref cat “family member” ^e^ref cat “married or in a partnership” ^f^ref cat “living alone” ^g^ref cat “poor/fair mental health” ^h^ref cat “no burden” ^i^ref cat “very well” ^j^ref cat “someone close to me” ^k^ref cat “no” ^l^ref cat “less than usual” ^m^ref cat “yes”. ^n^ref cat ”dementia”. 95% CI first column depicts min value; second column depicts max value.

**Section C. Models fit using residual distributions**

**Figure S3. Residual distribution for overall, emotional, and social loneliness**

| 1. Overall loneliness residuals |
| --- |
| 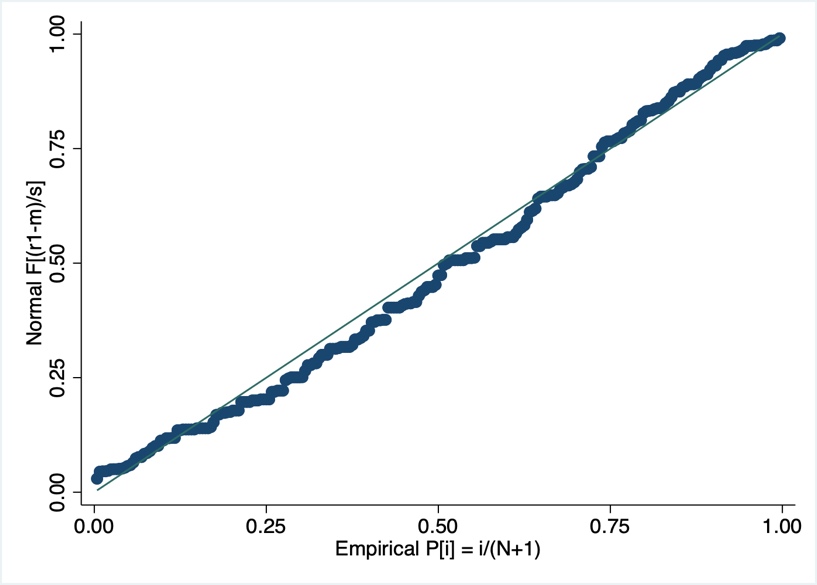 |
| 1. Emotional loneliness residuals |
| 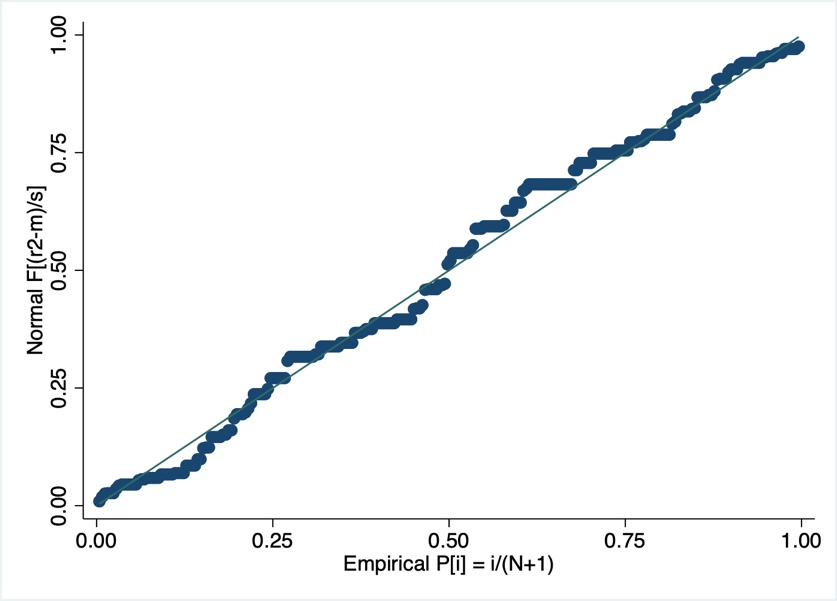 |
| 1. Social loneliness residuals |
| 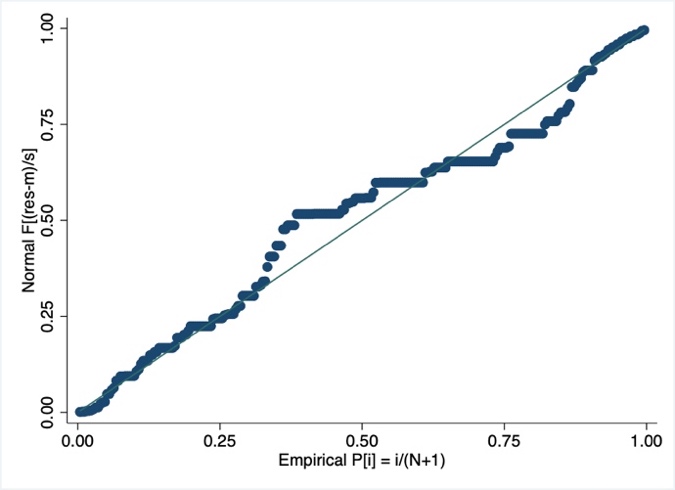 |
